# A Wind Tunnel Study of the Flow-Induced Vibrations of a Cylindrical Piezoelectric Transducer

**DOI:** 10.3390/s22093463

**Published:** 2022-05-02

**Authors:** Shehab Salem, Karel Fraňa

**Affiliations:** Department of Energy Equipment, Faculty of Mechanical Engineering, Technical University of Liberec, 461 17 Liberec, Czech Republic; karel.frana@tul.cz

**Keywords:** piezoelectric sensors, flow-induced vibration, energy harvesting, vortex shedding, cylinder wake

## Abstract

Piezoelectric transducers are used as a sensing device to study the fluids’ motion. Moreover, they are used as a harvester of energy of Flow-Induced Vibration (FIV). The current FIV harvesters in the literature rely on piezoelectric cantilevers coupled with a bluff body that creates flow instabilities. This paper studies the use of piezoelectric cylinders as a novel transducer in the field of fluid mechanics, where the transducer makes use of its bluff geometry to create instability. The study was based on wind tunnel measurements performed on four piezoelectric cylinders of different sizes over a speed range of 1–7 m/s. The paper looks at the variation of the generated voltage across the Reynolds number. It also compares the spectra of the generated open-circuit voltage to the turbulence spectra features known from the literature.

## 1. Introduction

Recently, piezoelectric materials have gained a broader role in the field of energy harvesting, where they are being used to develop Piezoelectric-Based Energy Harvesters (PBEH). Various energy harvesters have been developed to harvest the energy of mechanical vibrations in different configurations. Examples of such harvesters can be found in the reviews presented in [1,2].

In addition to mechanical vibration harvesting, various PBEHs have been developed to harvest flow energy using turbine-based harvesters. They rely on a turbine to extract the flow’s kinetic energy and transfer it into a piezoelectric transducer, where it is converted into electrical energy. Examples of such harvesters can be found in [3,4,5]. Moreover, PBEHs have been developed to harvest the energy of Flow-Induced Vibrations (FIV), where a bluff body is inserted into a flow, creating vortices. The vortices’ energy is then harvested by adding a PBEH that makes use of the variation in fluid pressure due to the turbulence.

FIV is a broad branch of vibrations that includes: Vortex-Induced Vibration (VIV), Flutter, and Galloping [6]. VIV is a form of a limited-amplitude vibration created by the loads induced during the vortex shedding off a bluff body. Galloping is a kind of vibration that depends on the variation in the flow angle of attack during the vibration. Therefore, it requires a non-symmetric bluff body—not a circular cylinder—to form. Finally, Flutter is a similar form of vibration to VIV, with the difference that it involves the interaction between the aerodynamic loads and the elastic deformation of the bluff body. Hence, it is a self-feeding vibration and is mainly found in plate-like bodies [7]. In the next section, VIV shall be discussed in more detail.

When a flow of a low Reynolds Number Re flows past a bluff body, a regime of vortices is formed in the body wake region. This regime induces periodic loads on the body, causing it to vibrate. As the Reynolds number increases, the nature of the vortex regime will change, causing an increase in the vortex shedding frequency f_s._ until the vortex train becomes fully turbulent and chaotic. If the vortices—and, hence, the loads—are formed with an equal frequency to the natural frequency of the transducer, the transducer goes through resonance in a state known as *Lock-in* [8]. The reader may refer to [9] as a review of the different vortex-shedding regimes.

The frequency of vortex shedding is defined by the Strouhal number St, which is the ratio between the inertial force of the vortices to the inertial force of the flow main stream [10]. Extensive work has been done to determine the value of Strouhal number. This work has resulted in several empirical models that relate Strouhal number to Reynolds number [11]. 

The process of vortex shedding, and its resultant VIV, have long been used in energy harvesting devices. Harvesting the energy of VIV started with harvesting the VIV from water flow [12]. Examples of such work could be found in [13,14], while work focused on harvesting VIV from airflow is presented in the next section. 

Pobering et al. [15] have developed a harvester composed of three piezoelectric bimorph cantilevers mounted behind a bluff body. The harvester was tested inside a wind tunnel at a wind speed of 4.5–45 m/s. The harvester generated a maximum voltage of 0.83 Volts at an airflow velocity of 35 m/s and generated power up to 108 μW at a velocity of 45 m/s. Gao et al. [16] presented a PBEH based on a piezoelectric cantilever coupled with a cylindrical extension. The cylinder in the cross-flow was free to vibrate and was directly attached to a piezoelectric device. The harvesters’ power output was found to increase with the wind velocity and cylinder diameter. Lee et al. [17] developed a PBEH composed of a cylindrical oscillator suspended on a silicon cantilever covered with a piezoelectric aluminum–nitride layer. The harvester was tested inside a wind tunnel alone, as well as with other dummy cylinders, in various configurations. It was found that increasing the number of turbulence shedding bodies—the dummy cylinders—from one to twenty-five increased the harvested power from 0.8 to 1.6 nW at a flow speed of 6 m/s. 

Akayidin et al. [18] designed a PBEH composed of a short piezoelectric polyvinylidene fluoride (PVDF) cantilever placed in the wake of a vortex-shedding cylinder. The harvester was tested inside a wind tunnel at an airflow speed of 7.23 m/s. This speed was chosen to ensure that the shedding frequency was matched to the harvester’s resonance frequency of 48.5 Hz. The harvester was able to generate 4 μW of power. Moreover, they tested the harvester with the cantilever placed at different distances from the cylinder, both span, and downstream-wise. They found that the harvester generated maximum power at an intermediate stream-wise distance from the cylinder. Either increasing or decreasing this distance would reduce the harvester output.

The only mention of an FIV energy harvester that was not based on a cantilever transducer was made by Xie et al. [19]. They numerically studied harvesting the energy of VIV via a cylindrical piezoelectric transducer immersed across an airflow of a speed of 5 m/s. The cylinder material was assumed to be PZT-5H and to have a resonance frequency of 98.5 Hz. The study was based on mathematical modeling of the energy harvesting process considering a model based on the piezoelectric constitutive equations, the flexural bending moment of a beam of a general profile, the equation of aerodynamic loads acting on a cylinder in a flow, and the equation of motion of the cylinder flexural vibration. They simulated cases of different load impedances, as well as cases of different load frequencies. Their results showed that maximum power was produced at a slightly higher frequency than the resonance frequency and at a load impedance that was five times larger than that of the cylinder. However, the maximum efficiency was produced at an equal load impedance to that of the cylinder.

According to the presented review, the current research has focused mainly on VIV energy harvesting that uses cantilever-configured transducers. The goal of this work is to study the applications of cylindrical piezoelectric transducers in the field of VIV energy harvesting. The paper investigates experimentally the response of cylindrically shaped transducers of different sizes to the energy existing in the airflow, considering the models found in the literature on the vortex shedding of circular cylinders. 

The investigation was performed by a series of wind tunnel measurements over an airspeed range of 1–7 m/s. The paper starts with an introduction section that discusses some of the characteristics of flow turbulence relevant to this study, as well as the recent trends in flow energy harvesting. Then, Section 2 of this paper describes the transducers understudy, the used experiment set-up, and the followed methodology. Section 3 presents the different results obtained from the measurements, while Section 4 discusses the results in the context of the known airflow characteristics and suggests a frame for future steps. Finally, Section 5 concludes the results of this paper. 

## 2. Materials and Methods

### 2.1. Materials

The four cylinders understudy are made from PIC 151, which is a modified lead Zirconate-Titanate (PZT-5A). The cylinders are provided by the supplier in specific sizes. The four cylinders, A, B, C, and D, have diameters, d, of 40, 20, 10, and 6.3 mm, respectively. The 40-mm cylinder has a length, L, of 40 mm, while the other cylinders have a length of 30 mm. These dimensions form an Aspect Ratio, as described in Table 1, ranging from 1 to 4.76. Unfortunately, it was not possible to control the cylinder aspect ratio. Finally, the four cylinders, A, B, C, and D, have an electric capacitance C of 70, 35, 36 and 18 nF, respectively. Table 1 summarizes the dimensions and the electric capacitance values of the four cylinders.

### 2.2. Description of the Experiment Set-Up

**Figure 1:**
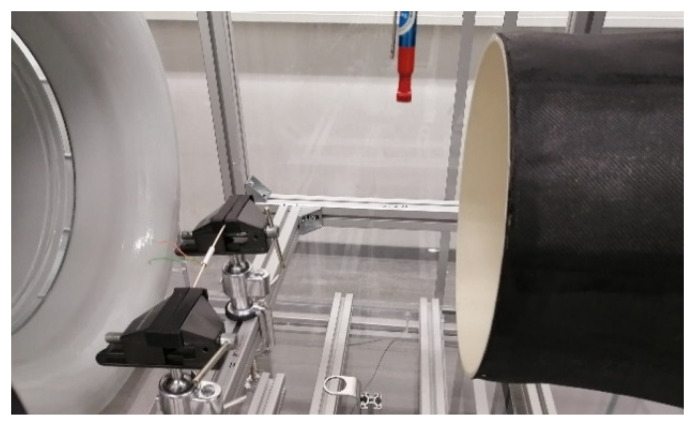
The cylinder is placed inside the tunnel test section perpendicular to the flow.

**Figure 2:**
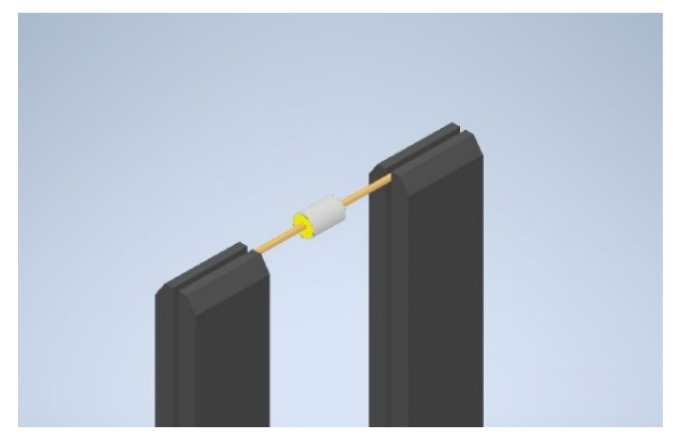
A 3D model of the fixation of the cylinder inside the wind tunnel test section.

The set-up was composed of a wind tunnel of the closed-circuit type. The wind tunnel has an operating wind speed in the range of 1–7 m/s. Inside the wind tunnel test section, a wooden beam was used as a mounting axis for the cylinders, as shown in Figure 1. The beam was clamped via two bench vises such that the cylinder axis was horizontal and perpendicular to the flow direction. The cylinder was mounted on the wooden beam by means of a sponge that was fitted between the inner surface of the cylinder and the wooden beam, as shown in Figure 2.

### 2.3. Methods

To study the response of the piezoelectric cylinders to airflow, each cylinder was subjected to an airflow of a defined speed U while measuring the open-circuit voltage V signal generated on the cylinder surface. The voltage was measured via the bench oscilloscope MSO-X 3054 A by Agilent Technologies with specifications as summarized in Table 2. For convenience, the oscilloscope probes were connected to the cylinder poles in such a way that a positive signal was obtained in response to the compressive loads from the airflow. Each signal was recorded for a duration of twenty seconds with a time resolution of 0.32 milliseconds. The oscilloscope vertical resolution was set to 10 mV/division, while the vertical offset was set to zero. The recorded time signals were used to calculate the Fast Fourier Transform (FFT) of each measurement case. The computed spectrum spanned over 1 kHz centered at 500 Hz with a spectral resolution Δf of 0.0153 Hz. Table 3 summarizes the data acquisition settings. 

The measurements were performed at wind speeds ranging from 1 to 7 m/s in steps of 1 m/s, with one additional point taken at a speed of 1.5 m/s. At each windspeed U for each cylinder of diameter d, it was possible to determine the Reynolds number Re of the flow using Equation (1), where the kinematic viscosity ν was assumed to be 1.56 × 10^−5^ m^2^/s. Then, it was possible to use Roshko’s model for the Strouhal number St at Re above 300 [20] to calculate the respective Strouhal number using Equation (2). Finally, it was possible to calculate the expected vortex shedding frequency f_s_ using Equation (3).

(1) Re= U dν

(2) St=0.212(1−12.7Re)

(3) $$f_{s}=\frac{\mathrm{St}\;U}{d}$$

## 3. Results

### 3.1. Time Response

**Figure 3:**
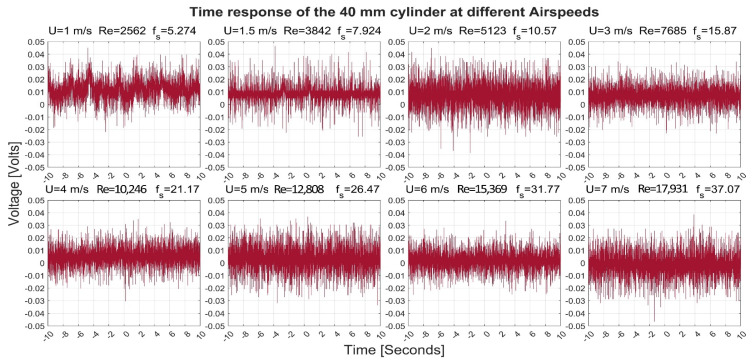
Time response of the 40-mm cylinder at different airflow speeds U. Re is the Reynolds number and f_s_ is the shedding frequency in Hz.

**Figure 4:**
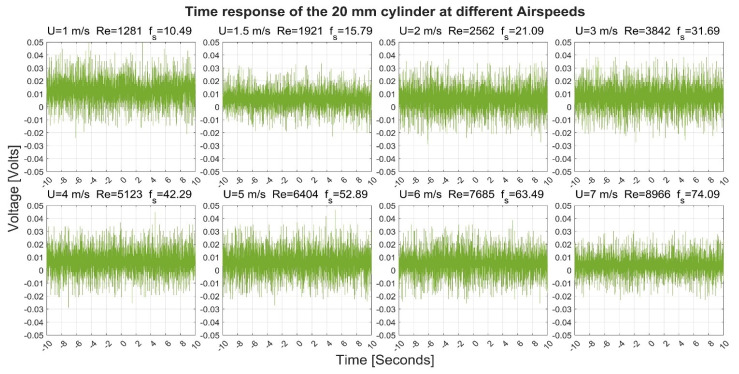
Time response of the 20-mm cylinder at different airflow speeds U. Re is the Reynolds number and f_s_ is the shedding frequency in Hz.

**Figure 5:**
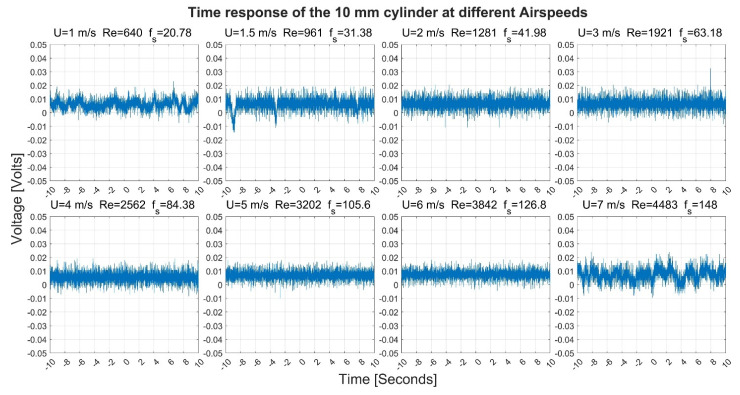
Time response of the 10 mm cylinder at different airflow speeds U. Re is the Reynolds number and f_s_ is the shedding frequency in Hz.

**Figure 6:**
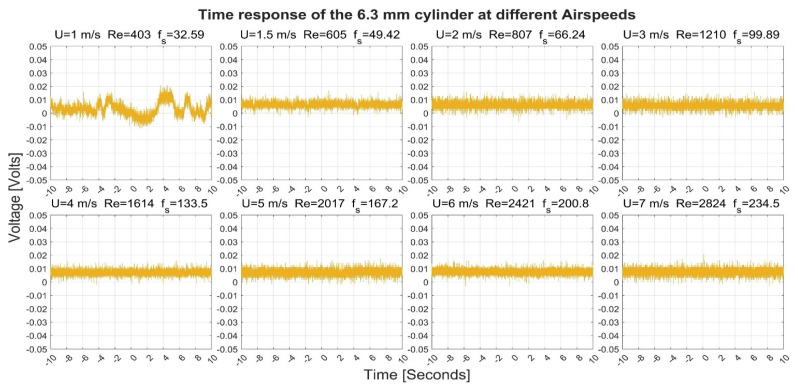
Time response of the 6.3-mm cylinder at different airflow speeds U. Re is the Reynolds number and f_s_ is the shedding frequency in Hz.

Figure 3, Figure 4, Figure 5 and Figure 6 show the time-response obtained from the 40 mm, 20 mm, 10 mm, and 6.3 mm cylinders, respectively, at different airflow speeds. The figures also indicate the shedding frequency f_s_ calculated using Equation (3). From these figures, it can be seen that the cylinders generated an oscillating voltage signal. Such oscillations are characteristic of the phenomenon of vortex shedding. For the 40-, 20-, and 6.3-mm cylinders, it was possible to find clear pulses in the signals at some flow speeds. To name a few examples, pulses were found in the signals from the three cylinders at a flow speed of 1 m/s. Moreover, the 10-mm cylinder gave interesting pulses at flow speeds of 1.5 and 7 m/s.

Moreover, the generated pulses spanned over a range that changed with the change in the airspeed. For example, the 40-mm cylinder pulses varied between −0.02 and 0.04 Volts at a speed of 1 m/s while it spanned between +/− 0.03 Volts at a speed of 7 m/s. For the 20-mm cylinder, its pulses varied between −0.02 and 0.05 at a speed of 1 m/s while it spanned between −0.02 and 0.03 Volts at a speed of 7 m/s

The 10- and 6.3-mm cylinders gave smaller pulses than the first two cylinders. The 10-mm cylinder gave pulses that varied between 0 and 0.02 Volts at a speed of 1 m/s while it spanned between −0.05 and 0.025 Volts at a speed of 7 m/s. The 6.3 mm cylinder gave pulses that varied between −0.01 and 0.02 at a speed of 1 m/s, while it spanned between 0 and 0.01 volts at a speed of 7 m/s. 

### 3.2. Effect of the Airflow Speed on the Cylinder Response

**Figure 7:**
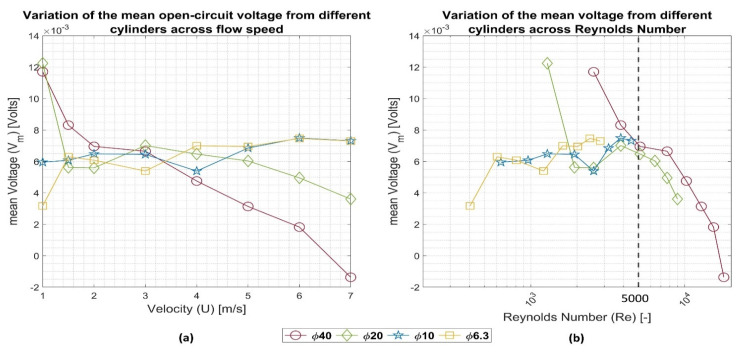
Variation in the mean voltage V_m_ from all cylinders across the (**a**) flow speed U and (**b**) Reynolds number Re. The black dashed line shows the onset of vortex shedding degradation according to [21].

Figure 7 shows the variation in the mean voltage over each of the airflow speeds and the Reynolds number for the four cylinders. By performing the measurements on cylinders of various diameters over the same speed range, it was possible to demonstrate the energy transfer behavior over a wide range of Reynolds numbers, as shown in Figure 7b. The lower range of Reynolds numbers was demonstrated using the smaller sensor diameters, while the larger range was demonstrated using the larger sensor diameters.

From Figure 7a, it can be seen that the 40- and the 20-mm cylinders tended to generate more voltage at a lower flow speed—a less turbulent flow—generating a maximum voltage of 11.7 and 12.2 mV, respectively, at a flow speed of 1 m/s. However, as the velocity increased, the two cylinders’ output dropped, generating minima of 3.6 and −1.3 mV, respectively, at a flow speed of 7 m/s. The drop rate of the mean of the two cylinders changed over airspeeds of 1.5–3 m/s, where the 40-mm cylinder drop decelerated over the range mentioned above while the 20-mm cylinder drop even rose before dropping again at speeds above 3 m/s. 

The cylinders’ behavior can be interpreted differently across the Reynolds number scale, as shown in Figure 7b. It can be seen that the two cylinders’ voltages tended to decline with the increase in Reynolds number, with an exception for the 20-mm cylinder in the range 2000 < Re < 4000—which corresponds to 1.5 < U < 3 m/s—which is the transitional region between the laminar and turbulent flow. Unfortunately, it was not possible to test these two cylinders in the laminar range of the Reynolds number scale, since these cylinders’ sizes would require lower velocities than those that were rated for the wind tunnel operation. Finally, it was most interesting that the two cylinders showed an identical and substantial voltage drop rate at Re > 4000.

Considering the 10- and 6.3-mm cylinders, it was found that they showed different behaviors to the first two cylinders. The cylinders’ mean voltage tended to increase with the increase in the flow speed, with one exception for each cylinder. The 10-mm cylinder voltage dropped at a speed of 4 m/s—Reynolds number of 2562, while the 6.3-mm cylinder voltage dropped over the speed range 1.5–3 m/s—Reynolds number of 600–1200. Finally, it is important to point out that it is expected that the cylinder size will play a role in interaction with the flow turbulence according to the relationship between the cylinder diameter and the turbulence scale.

The drop in the generated voltage at Re > 4000 was found to agree with the findings of Norberg [21]. He showed that the vortex shedding is of high quality over the range 260 < Re < 5000, while it changes its appearance over 5000 < Re < 2 × 10^5^, where it becomes broader due to the change in shedding frequency over time. Therefore, a vertical dashed line is plotted in Figure 7b at Re = 5000. The mean voltage drops past this line. 

### 3.3. Frequency Response

**Figure 8:**
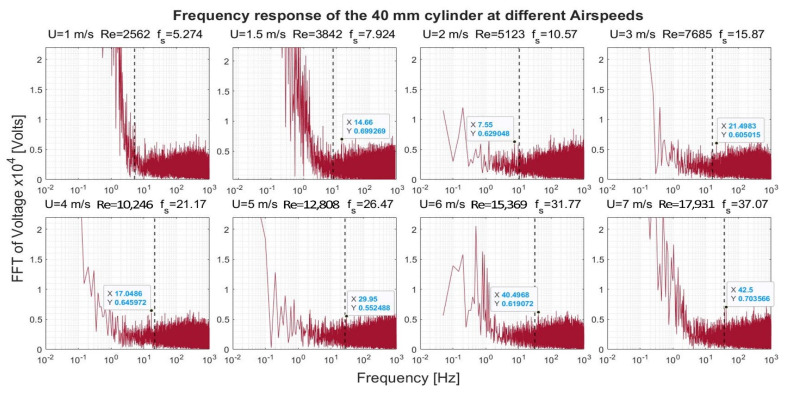
Frequency response of the 40-mm cylinder at different airflow speeds U. Re is the Reynolds number, and f_s_ is the shedding frequency in Hz, indicated by a black dashed line.

**Figure 9:**
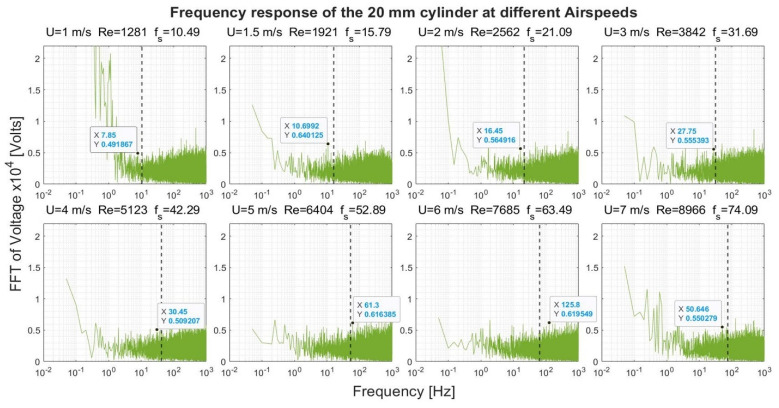
Frequency response of the 20-mm cylinder at different airflow speeds U. Re is the Reynolds number, and f_s_ is the shedding frequency in Hz, indicated by a black dashed line.

**Figure 10:**
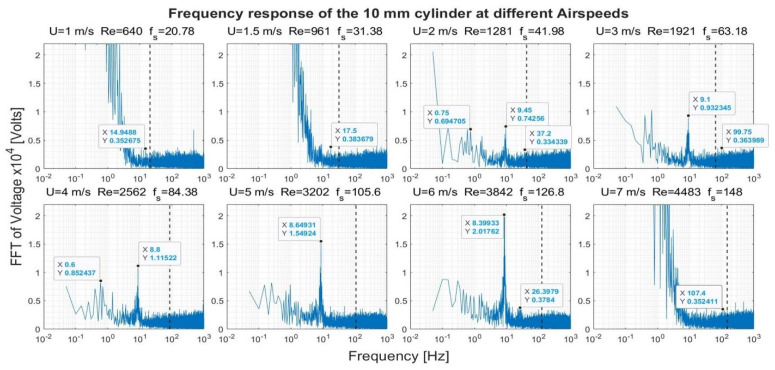
Frequency response of the 10-mm cylinder at different airflow speeds U. Re is the Reynolds number, and f_s_ is the shedding frequency in Hz, indicated by a black dashed line.

**Figure 11:**
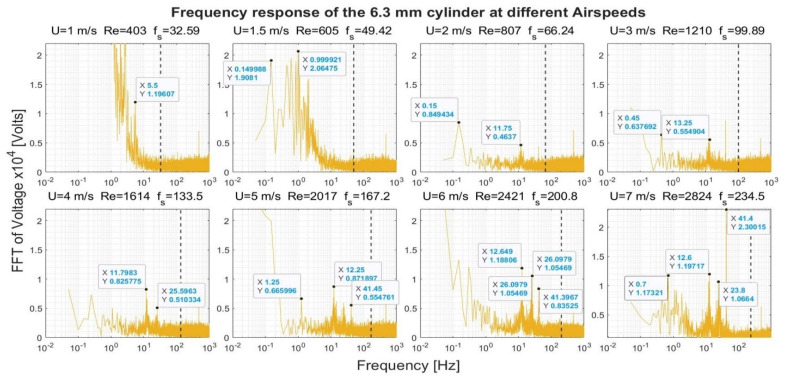
Frequency response of the 6.3-mm cylinder at different airflow speeds U. Re is the Reynolds number, and f_s_ is the shedding frequency in Hz, indicated by a black dashed line.

Figure 8, Figure 9, Figure 10 and Figure 11 show the frequency response that was obtained from the four cylinders. In these figures, a black vertical dashed line was plotted to indicate the expected shedding frequency. The voltage FFT extended over a wide range—thousands-fold, as will be shown later in Section 4.1.2. The present section will be focused on the range up to 2.2 × 10^−4^, where some interesting voltage peaks could be found. Figure 8 and Figure 9 show the generated frequency spectra from the 40-mm and 20-mm cylinders, respectively, on a linear vertical scale. From these two cylinders, it was interesting to find some significant broadband peaks that matched the shedding frequencies f_s_ calculated from Equation (3). However, no significant peaks were found from the 40-mm cylinder at 1 m/s airflow speed.

Figure 10 and Figure 11 show the frequency spectra of the signals from the 10- and 6.3-mm cylinders, respectively, at different airflow speeds. Like the previous two cylinders, it was possible to identify some frequency broadbands that matched the frequencies calculated from Equation (3). Moreover, it was interesting to find that high-quality peaks were formed at other, lower frequencies. To name a few examples, the 10-mm cylinder formed a dominant peak in Figure 10 over the range 8.4–9.45 Hz. The peak started to appear at speeds of 2–6 m/s; however, it disappeared at speed of 7 m/s. The 6.3-mm cylinder also formed a dominant peak in Figure 11 across the range of 11.5–13.5 Hz. The peak appeared at speeds of 2–7 m/s. Moreover, other neighboring dominant peaks started to form at a speed of 4 m/s. It is important to mention that these dominant peaks from the two latter cylinders do not match the calculated shedding frequencies. It is possible that these peaks were created by the cylinder support. 

## 4. Discussion

### 4.1. Analogy between the Vortex Shedding and Piezoelectric Transduction

#### 4.1.1. Mean Generated Voltage versus Quality of Vortex Shedding

It was expected that the energy content of the airflow—and, hence, the cylinder open-circuit voltage—would be proportional to the second power of airspeed. However, the data in Figure 7a showed otherwise. It was difficult to find a general trend for the output voltage over airspeed. However, more clear trends in the voltage over Reynolds number were found in Figure 7b, where it was clearly shown that the generated voltage drops at Re > 4000. This drop was confirmed by two cylinders, the 40-mm and the 20-mm cylinders, and agreed with other independently measured data of vortex-shedding off cylinders. This agreement may indicate an analogy between the fluid phenomena and the piezoelectric transduction—at least for the 40- and 20-mm cylinders. Moreover, it proved that the critical parameter for VIV harvesting is the Reynolds’ number rather than the airspeed. 

#### 4.1.2. Frequency Response

**Figure 12:**
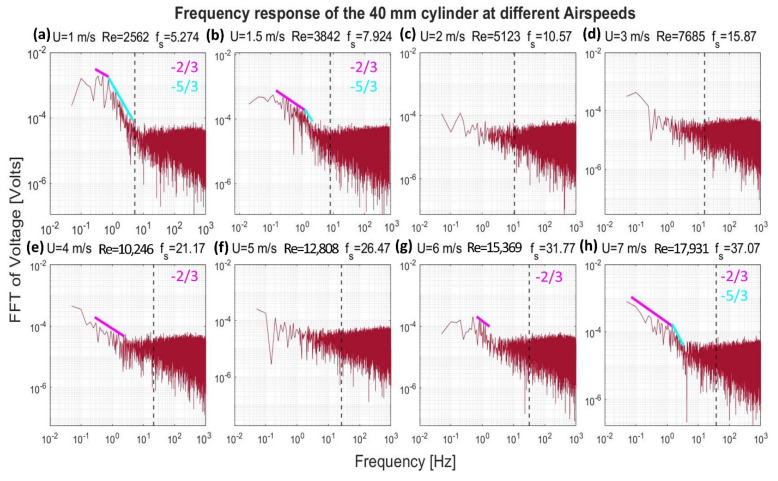
Frequency response of the 40-mm cylinder in logarithmic scale at different airflow speeds U. Re is the Reynolds number, and f_s_ is the shedding frequency in Hz, indicated by a black dashed line.

**Figure 13:**
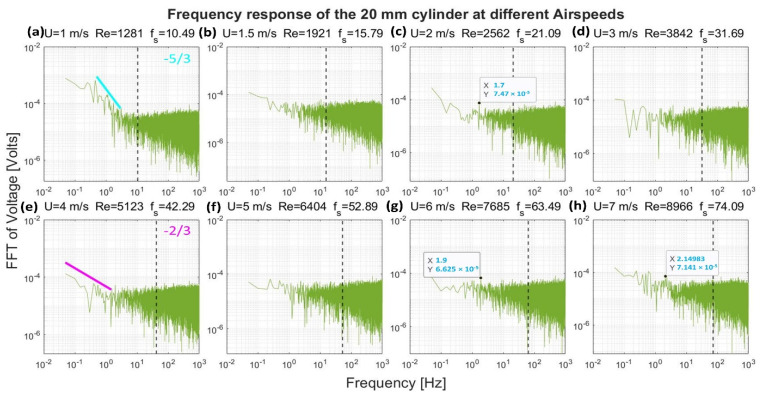
Frequency response of the 20-mm cylinder in logarithmic scale at different airflow speeds U. Re is the Reynolds number, and f_s_ is the shedding frequency in Hz, indicated by a black dashed line.

**Figure 14:**
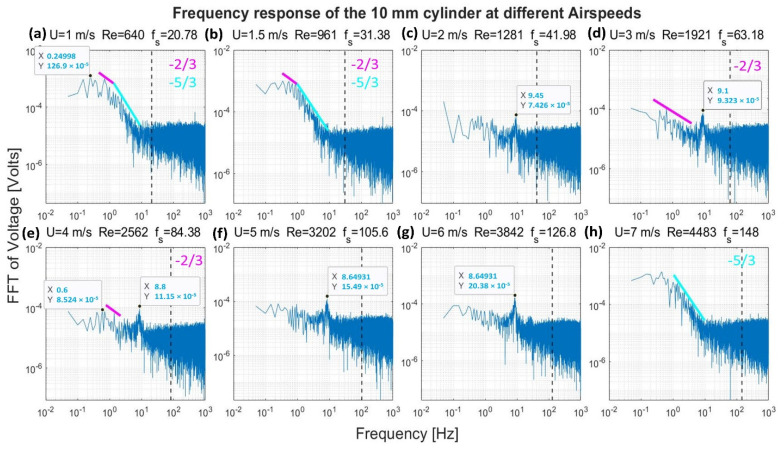
Frequency response of the 10-mm cylinder in logarithmic scale at different airflow speeds U. Re is the Reynolds number, and f_s_ is the shedding frequency in Hz, indicated by a black dashed line.

**Figure 15:**
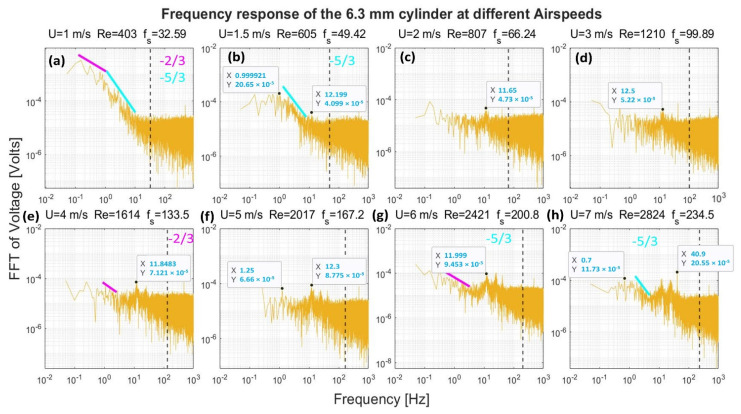
Frequency response of the 6.3-mm cylinder in logarithmic scale at different airflow speeds U. Re is the Reynolds number, and f_s_ is the shedding frequency in Hz, indicated by a black dashed line.

Figure 12, Figure 13, Figure 14 and Figure 15 show, in a logarithmic scale, the generated frequency spectra shown before, together with lines indicating a logarithmic drop rate of five-thirds and two-thirds plotted above the regions of the FFT that had such a drop rate.

Figure 12 shows the response of the 40-mm cylinder. For flows of 1 m/s, the strength of the FFT followed a two-thirds logarithmic drop rate from the lowest frequency up to 1 Hz. From 1–5 Hz, the drop rate was five-thirds. The same behavior was found at flows of 1.5 m/s and 7 m/s, with a transition from the two-thirds rate to the five-thirds rate that occurred at a higher frequency. A two-thirds drop rate was captured over some frequency ranges for the 4 m/s and 6 m/s airflow speeds. For the 20-mm cylinder, shown in Figure 13, it was only possible to capture the five-thirds rate in the case of the 1 m/s speed, while a two-thirds rate was captured for the 4 m/s speed. Moreover, an interesting broadband peak was found at frequencies of 1.7–2.2 Hz at different speeds.

For the 10-mm cylinder shown in Figure 14, the two drop rates were found together at speeds of 1 and 1.5 m/s. Moreover, a two-thirds rate was found at 3 m/s and 4 m/s flow speeds, while a five-thirds rate was found at 7 m/s. Finally, Figure 15 shows the frequency spectrum from the 6.3 mm cylinder. Both the drop rates—the two-thirds and the five-thirds—were found together in case of the 1 m/s and 1.5 m/s speeds. The two-thirds rate was found at a speed of 3 m/s and 4 m/s, while the five-thirds rate was found in the 7 m/s only.

From the previous discussion, it is evident that it was always possible to find a five-thirds linear drop rate at a speed of 1 m/s—the most laminar measurement for each cylinder—from all cylinders. It was even possible to find this rate at other flow velocities. This linear drop—being plotted on a logarithmic plot versus frequency—indicates that voltage V is distributed across its frequency f according to a power law of order of negative five-thirds as summarized by Equation (4), where Const. is a proportionality constant. Assuming that the generated voltage V corresponds to the turbulence energy of the flow E and that the frequency f is analogous to the wavenumber k, this model is found to resemble the model established by Kolmogorov to describe a turbulent flow in the inertial range of the wavenumber and how its different turbulence scales interact and cascade the energy from the larger scales to the smaller ones [22]. His model was described by Equation (5), which relates the spectral energy E(k) of each wave number k to the respective wavenumber and the turbulence dissipation rate ε only where α is another proportionality constant. Comparing Equations (4) and (5), the level of resemblance between the piezoelectric transduction and fluid phenomena is clear.

(4) $$V\left(f\right)=\mathrm{Const}.\times {\left(f\right)}^{\frac{-5}{3}}$$

(5) $$E\left(k\right)=\alpha .\varepsilon^{\frac{-2}{3}}\times {\left(k\right)}^{\frac{-5}{3}}$$

### 4.2. Variation in Charge Generation across Reynolds Number

Piezoelectric transducers respond to mechanical loads by forming electric charges. According to the transducer’s electrical capacitance, the electric charges will form an electric potential. Therefore, it would be interesting to look at the energy transfer process—from mechanical to electrical—using the above data, normalized to the individual cylinder characteristics.

**Figure 16:**
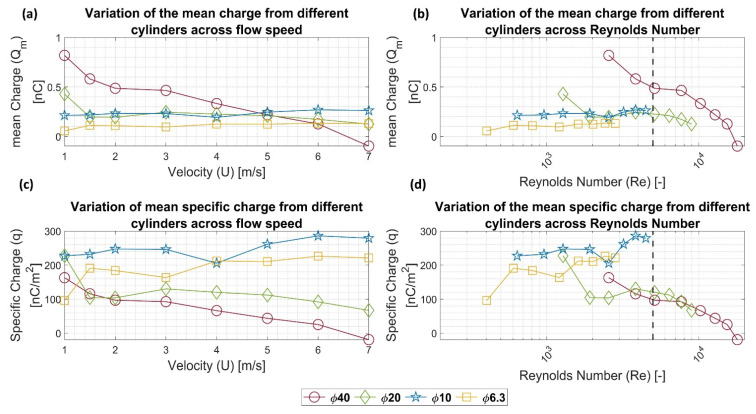
The variation in the mean total charge Q across (**a**) the flow speed, and (**b**) Reynolds number Re based on cylinder diameter for all cylinders. The variation in the specific charge q across the (**c**) flow speed, and (**d**) Reynolds number Re based on cylinder diameter for all cylinders. The black dashed line in (**b**,**d**) shows the onset of vortex shedding degradation according to [21].

To exclude the effect of the individual cylinder—the cylinder surface area and the capacitance—on the generated signal, the total electric charge Q and the specific electric charge q—charge per unit cylinder surface area—were calculated using Equations (6) and (7), respectively, and were plotted across flow speed and Reynolds number in Figure 16a,b, respectively. Due to the linear relation between the electric charge and the voltage, the electric charge had a similar trend across speed to that of the mean voltage, as shown in Figure 16a. Moreover, the data in Figure 16a show that more charges were generated from the cylinder with the higher diameter. This behavior prevailed up to a speed of 5 m/s. Above this speed, the 10-mm cylinder had the highest charge. In the sub-five meter per second range, the 10- and 20-mm cylinders had almost equal charge values. It is worth mentioning that the difference between their capacitances is 1 nF, as indicated in Table 1. The data appear more clearly over the Reynolds number scale, as plotted in Figure 16b.

Figure 16c,d show the variation in the specific electric charge over the flow speed and Reynolds number, respectively. In Figure 16c, it was interesting to find that the 10-mm cylinder had the highest specific charge generation among all cylinders. Moreover, charge generation had two peaks, at 2 m/s and 6 m/s, which corresponds to Reynolds numbers of 1281 and 3842, respectively. By plotting these data across the Reynolds number range in Figure 16d, it was interesting to find that the 40-mm and 20-mm cylinders achieved continuity in the specific charge at Reynolds number ranges above 4000, while such continuity was missing for the 10- and 6.3-mm cylinders. Again like Figure 7b,a black dashed line indicates in Figure 16b,d the onset of vortex-shedding degradation according to Norberg [21].

(6) $$Q_{m}={\mathrm{CV}}_{m}$$

(7) $$q=\frac{Q_{m}}{\pi \;d\;L}$$

### 4.3. The Mode of Turbulence

It is evident from Figure 12, Figure 13, Figure 14 and Figure 15 that the peaks centered around the shedding frequencies were at a lower order of magnitude (1000 times less) than the dominant frequencies in the spectra. This may be because vortex shedding was not the main contributor to energy generation in the measurements, contrary to other piezoelectric VIV harvesters that operate on vortex shedding using a cylindrical extension—a non-piezoelectric one. This contradiction may be justified by the fact that the transducer in these harvesters is placed at a certain distance behind the cylindrical extension. This distance allows for the vortices to develop before interacting with the transducer. Therefore, the transducer will have a dominant peak at the shedding frequency.

In the case of the cylinders understudy, the transducer—the cylinder—transduces the vortices effect before the vortices go through their formation length and develop. Moreover, the transducer signal output will be affected by its upstream wake region, contrary to other standard harvesters. Hence, the transducer is mainly affected by the wake of its cylindrical profile rather than vortex shedding.

### 4.4. The Effect of Cylinder Support

As was previously mentioned in Section 2.2, the cylinders were mounted on a wooden stick that was fixed via two bench vises at its ends. Such a structure can be modeled as a simply supported beam subjected to a point load at its middle, where this load is the cylinder weight. The structure will have a natural frequency that depends on the beam profile geometry, the beam length, the cylinder weight, and the beam material.

The natural frequency of the structure can be exploited in two opposite ways. In sensor-based applications, it would be necessary to design the structure to have a natural frequency outside of the measurement range. On the other hand, in FIV energy harvesting, it would be necessary to design the structure to achieve a natural frequency identical to the dominant frequency in the flow to achieve the Lock-in condition.

### 4.5. Future Work

The work presented in this paper discussed the interaction between cylindrical piezoelectric transducers and flow characteristics over a wide Reynolds number range. The wide range was achieved using different cylinders at different airflow speeds. It would be interesting to extend the study on each cylinder over a wider speed range to compare different cylinder sizes at the same Reynolds number. Moreover, it would be interesting to extend the study over a lower Reynolds number range than the one used in this paper. Special attention should be given to the range at 150 < Re < 400 in order to compare the response to regular and irregular vortex trains. Finally, future work should consider the design of the mounting structure to ensure that its natural frequency lies outside of the targeted measurement range.

## 5. Conclusions

This paper tackled the cross-over between the flow characteristics across circular cylinders and piezoelectric materials. The paper investigated the response of cylindrical piezoelectric transducers of four different sizes placed across the airflow to the well-known behavior of a flow across a cylinder at different airflow speeds. The investigation focused on the variation in the electric charge generation over Reynolds number, as well as the turbulence spectrum of flow across circular cylinders. Measurements were performed in the laminar, transitional, and subcritical regions of the Reynolds number scale in the range 403–17,931. Regarding the behavior of electric charge generation, it was possible to find a resemblance between the data obtained from the two cylinders of diameters 40 and 20 mm, in addition to the similarity between the data obtained from the two cylinders of diameters 10 and 6.3 mm. Regarding the vortex shedding frequency, it was possible to locate broadband peak frequencies in the measured data near the calculated vortex shedding frequencies. However, these peaks were not the strongest peak in the signal, indicating that the vortex shedding was not the only turbulence mode in the flow. 

## Figures and Tables

**Table 1:** Characteristics of the piezoelectric cylinders.

Item	Cylinder A	Cylinder B	Cylinder C	Cylinder D
Diameter d (mm)	40	20	10	6.3
Length L (mm)	40	30	30	30
Aspect Ratio L/d (-)	1	1.5	3	4.76
Electric Capacitance C (nF)	70	35	36	18

**Table 2:** Oscilloscope Accuracy Settings.

Parameter	Value	Error Margin (mV)
Number of vertical divisions	10	-
DC vertical gain accuracy	±2.0% full scale	±2
DC vertical offset accuracy	±0.1 div ± 2 mV ± 1% of offset setting	±3

**Table 3:** Signal Acquisition Settings.

Signal Duration (s)	Vertical Precision (mV/div.)	Temporal Resolution (ms)	FFT Spectral Resolution (Hz)	FFT Frequency Span (Hz)
20	10	0.32	0.0153	1000

## Data Availability

All data are available from the authors on request.
